# Laser-Induced Periodic Surface Structuring of Poly(trimethylene terephthalate) Films Containing Tungsten Disulfide Nanotubes

**DOI:** 10.3390/polym12051090

**Published:** 2020-05-10

**Authors:** Javier Prada-Rodrigo, René I. Rodríguez-Beltrán, Sandra Paszkiewicz, Anna Szymczyk, Tiberio A. Ezquerra, Pablo Moreno, Esther Rebollar

**Affiliations:** 1 Grupo de Aplicaciones del Láser y la Fotónica (ALF-USAL), Universidad de Salamanca, Pl. de la Merced s/n, 37008 Salamanca, Spain; rrodrigu@cicese.mx (R.I.R.-B.); pmoreno@usal.es (P.M.); 2 Instituto de Química Física Rocasolano, Consejo Superior de Investigaciones Científicas (IQFR-CSIC), Serrano 119, 28006 Madrid, Spain; 3 CONACYT-Unidad Foránea Monterrey, Centro de Investigación Científica y de Educación Superior de Ensenada, Alianza Centro 504, PIIT, Apodaca, Nuevo León CP 66629, Mexico; 4 Department of Materials Technology, Faculty of Mechanical Engineering and Mechatronics, West Pomeranian University of Technology, Piastow Av. 19, PL-70310 Szczecin, Poland; spaszkiewicz@zut.edu.pl; 5 Department of Technical Physics, Faculty of Mechanical Engineering and Mechatronics, West Pomeranian University of Technology, Piastow Av. 19, PL-70310 Szczecin, Poland; anna.szymczyk@zut.edu.pl; 6 Instituto de Estructura de la Materia, Consejo Superior de Investigaciones Científicas (IEM-CSIC), Serrano 121, 28006 Madrid, Spain; t.ezquerra@csic.es

**Keywords:** LIPSS, nanostructures, nanocomposites, laser material processing, ultrashort pulses

## Abstract

We report the study of the formation of Laser Induced Periodic Surface Structures (LIPSS), with UV femtosecond laser pulses (λ = 265 nm), in free-standing films of both Poly(trimethylene terephthalate) (PTT) and the composite PTT/tungsten disulfide inorganic nanotubes (PTT-WS_2_). We characterized the range of fluences and number of pulses necessary to induce LIPSS formation and measured the topography of the samples by Atomic Force Microscopy, the change in surface energy and contact angle using the sessile drop technique, and the modification in both Young’s modulus and adhesion force values with Peak Force-Quantitative Nanomechanical Mapping. LIPSS appeared parallel to the laser polarization with a period close to its wavelength in a narrow fluence and number of pulses regime, with PTT-WS_2_ needing slightly larger fluence than raw PTT due to its higher crystallinity and heat diffusion. Little change was found in the total surface energy of the samples, but there was a radical increase in the negative polar component (*γ−*). Besides, we measured small variations in the samples Young’s modulus after LIPSS formation whereas adhesion is reduced by a factor of four. This reduction, as well as the increase in *γ−*, is a result of the modification of the surface chemistry, in particular a slight oxidation, during irradiation.

## 1. Introduction

Polymers are widely used in many different fields due to their versatility and reasonable price-performance ratio. However, some specific applications, such as their use as biomaterials [1], protective coatings [2], and in thin film technology to name a few, require polymers with enhanced properties. Different approaches have been developed to address that need. In this work, we use two of them: (i) fabrication of nanocomposites by in-situ polymerization [3,4], where a small amount of a nanomaterial is added to a polymeric matrix giving the resulting material some of the remarkable properties of the nanomaterial; and (ii) surface nanostructuring, where nanostructures are generated on the surface of a material, which can be engineered to change wettability, adhesion, surface energy, tribological properties and other physicochemical properties [5,6].

Regarding polymer nanocomposites, one can find applications in electronics, packaging, biotechnology and many others [1,2,7]. They are lighter than conventional composites, and their properties can be tailored by changing the additive and the polymer used, the percentage of additive, and the diffusion of the additive in the polymeric matrix [3].

In this work, Poly(trimethylene terephthalate) (PTT) was used both as a stand-alone material and as a matrix for our nanocomposite. PTT is an interesting material to use as a polymer matrix because of its crystallization rate, and the variety of ways it can be processed (injection molding, film casting and film spinning) [8]. Moreover, it can be fabricated out of a high percentage of renewable materials [9]. Tungsten disulfide (WS_2_) inorganic nanotubes were used as the additive of the nanocomposite. They are an alternative additive to carbon nanotubes to manufacture nanocomposites. Among their advantages is the low manufacturing expense, since catalysts are not needed for their fabrication and the precursors are relatively inexpensive [10]. Besides, they present high impact resistance and good tribological behavior. It is because of these properties that they have been applied successfully as reinforcing agents and to improve the tribological properties of a variety of materials [9,11].

As for the generation of surface nanostructures, it is a common practice employed to further improve the surface properties of any material and provide it with some degree of functionality. Although lithographic techniques [12,13] are the usual method to achieve this, there has been a growing interest in laser processing techniques [14,15,16,17], specifically, in Laser Induced Periodic Surface Structures (LIPSS) generation [18,19,20], due to its versatility, cleanliness and simplicity from the point of view of equipment requirements and operation. 

LIPSS are ripples that appear on the surface of materials after being irradiated with a laser under specific processing conditions, whose period is in the order of magnitude of the laser wavelength. Birnbaum [21] was the first to observe them at the bottom of an ablation crater produced with a ruby laser, and Sipe et al. later developed a first principle theory [22,23,24] explaining their origin. According to this theory, the origin of LIPSS is the modulation of the intensity of the laser produced by the interference of the incident wave with a surface wave, formed by scattering on the rough surface of the material, and their period can be calculated as:

(1) $$\Lambda =\frac{\lambda }{n_{ef}\pm sin\theta }$$

where λ is the laser wavelength, $n_{ef}$ is the effective refractive index at the selvedge region [25] and θ is the angle of incidence of the laser [26].

LIPSS formation has been reported in metals [27], dielectrics [28] and semiconductors [29]. Moreover, the dependence of the LIPSS period and depth with fluence, number of pulses and laser polarization was studied in several works [30,31,32].

Remarkably, LIPSS in polymers form below the ablation threshold fluence. The nanostructures are not formed as a result of material removal or ablation but following a reordering of the material on the surface. In amorphous polymers, LIPSS arise when the surface of the polymer is heated above the glass transition temperature (T_g_). At this temperature, its viscosity greatly diminishes allowing the polymeric chains to flow. In contrast, in crystalline polymers, the surface must reach the melting temperature to break the crystalline structure and be able to flow. Many polymers, and in particular PTT, are not crystalline but semicrystalline, which means that only a percentage of them is in a crystalline phase [33,34]. The higher this percentage, the higher the threshold temperature that allows the polymer to flow, and, therefore, the higher the fluence and number of pulses necessary for LIPSS formation [19]. 

In this work, we generated LIPSS with femtosecond laser pulses. fs-LIPSS are interesting as far as nonlinear absorption is the key phenomenon behind energy transfer to the material and it depends in a far less decisive way on wavelength compared to linear absorption. This provides more freedom when choosing the irradiation wavelength and thus allows to produce LIPSS with a period on demand. Moreover, since the interaction is much faster than the thermal relaxation times, all the phenomenology is dominated by the laser-matter interaction, and the thermally affected area is much smaller than with longer pulses, which enhances our control over the LIPSS area. Despite these advantages, few articles are dedicated to fs-LIPSS [35,36,37,38,39,40] and picosecond LIPSS [41,42] generation on polymers, in contrast with the abundant information on their generation with nanosecond pulses [19,43,44,45,46,47,48]. 

The formation mechanisms of fs-LIPSS are not yet fully understood. Bonse et al. tried to explain fs-LIPSS by assuming a variation in the refraction index of the material in Sipe’s theory, following the Drude model, creating the so-called Sipe–Drude model [30]. However, this model does not explain all the phenomena present in fs-LIPSS, such as High Spatial Frequency LIPSS (HSFL)—LIPSS that have a period smaller than laser wavelength and are aligned perpendicular to the regular LIPSS, also called Low Spatial Frequency LIPSS (LSFL). New theoretical models have been proposed to explain these phenomena, including a model based on self-organization from highly electrostatic instabilities originated by the laser [49], one based on thin-films hydrodynamics [50], one based on the analysis of an electronic excitation when short-lived plasma is created [51,52] or another model that uses Finite Differences Time Domain methods to solve Maxwell’s curl equations for linear, isotropic, dispersive materials with no magnetic losses [53].

The objective of this work is to modify the surface properties of PTT and the composite PTT/tungsten disulfide inorganic nanotubes (PTT-WS_2_) by means of LIPSS formation with a femtosecond laser and measure the triggered physicochemical changes. In this way, we can shed light on whether the creation of this nanocomposite and further LIPSS generation on the surface may be used together to enhance the properties of PTT and to check if LIPSS formation is as effective in PTT-WS_2_ as in raw PTT.

## 2. Materials and Methods 

### 2.1. Materials

The samples investigated are free-standing films, 200 ± 10 μm thick, of PTT and PTT-WS_2_ nanocomposite, prepared by in situ polymerization. The nanocomposite is made out of PTT as the matrix and WS_2_ nanotubes as the additive (0.5% in weight). The preparation procedure and characterization of the samples have been reported in previous work [9]. The more relevant properties for our work given there are shown in Table 1 and UV-Vis absorption spectrum of PTT is shown in Figure S1.

### 2.2. Laser Irradiation

All the irradiations were carried out in air with the third harmonic of a femtosecond laser system. This system consists of a Ti:Sa oscillator (Tsunami, Spectra Physics^®^, Mountain View, CA, USA) and a regenerative amplifier (Spitfire, Spectra Physics^®^, Mountain View, CA, USA). The output pulses have 260 fs (FWHM), λ = 265nm, a repetition rate of 1 kHz, an energy up to 1mJ and are linearly polarized. 

Regarding the irradiation set-up, the fs laser beam is focused perpendicularly on the surface of the sample, which is placed on a motorized XYZ translation stage. To control the number of pulses we used an electromechanical shutter, and to control the fluence we use neutral filters for coarse tuning as well as a λ/2 plate and a linear polarizer for fine tuning. A thermopile detector (407 A, Spectra Physics^®^, Mountain View, CA, USA) was used to measure the average power of the beam. We calculated the fluence from this average power measurements assuming the laser transversal mode is Gaussian TEM00, which is a reasonable approximation for this system.

For this study, we irradiated the sample varying the number of pulses at fluences per pulse ranging from 8.5 to 33.9 mJ/cm^2^.

### 2.3. AFM Measurements. Topography

Atomic Force Microscopy (AFM) technique was chosen to measure the surface topography. Due to the soft nature of our samples, we measured in tapping mode using an AFM Multimode 8 (Bruker^®^, Karlsruhe, Germany) system with the controller Nanoscope V (Bruker^®^) and the software Nanoscope Analysis 1.50 (Bruker^®^) for image analysis. The tips were silicon NSG30 (NT-MDT^®^) with a curvature radius of ~6 nm, a nominal resonant frequency of 320 kHz and a typical spring constant of 40 N/m. 

### 2.4. Contact Angle Measurements. Surface Energy

Intending to determine the changes in wettability and surface energy of the samples, we measured their contact angle (CA) with different liquids both before and after irradiation. The contact angle is the angle formed by the liquid-solid and the air-liquid interfaces when a drop is deposited in a planar solid. It gives a measure of the wettability of the material. Moreover, if we measure the contact angle of liquids of known surface energy in our material, we can obtain the components of the surface energy using the Owens, Wendt, Rabel and Kaelble (OWRK) model, which separates the total surface energy ($\gamma^{TOT}$) in a polar component ($\gamma^{p}$, due to acid-base interactions) and a dispersive component ($\gamma^{d}$, due to Lifshitz-van der Waals forces) [55,56], or the van Oss, Chaudhury and Good model, which further separates the polar component in a polar negative component ($\gamma^{-}$, associated with electron donors) and a polar positive component ($\gamma^{+}$, associated with electron acceptors) [57]. This last method requires the use of at least three liquids of different nature (apolar and polar). We used paraffin oil as the apolar liquid and deionized water and glycerol as the polar ones (surface energy components detailed in Table A1 [58]). 

The measurements were performed at room temperature and ambient humidity, by means of the sessile drop technique using a pocket goniometer PG2 (FIBRO system, Stockholm, Sweden). We carried out eight measurements for every different sample-liquid pair for non-irradiated samples and samples irradiated with 5000 pulses at 20.3 mJ/cm^2^, since preliminary AFM measurements guaranteed that those laser irradiation conditions generated well-ordered LIPSS.

### 2.5. PF-QNM Measurements. Adhesion and Elastic Modulus

Peak Force-Quantitative Nanomechanical Mapping (PF-QNM) [59] is a protocol developed by Bruker^®^, based on AFM, that measures the adhesion, elastic modulus, deformation and topography of a surface simultaneously, with the spatial resolution provided by AFM. This protocol was used to characterize the nanomechanical properties of the samples. Specifically, the surface elastic modulus was obtained by application of the Derjaguin–Muller–Toporov (DMT) model [60]:

(2) $$F-F_{adh}=\frac{4}{3}E^{*}R^{1/2}d^{3/2}$$

where *F* is the force applied to the scanning tip, *F_adh_* is the adhesion force between the tip and the sample, *R* is the tip radius, d is a parameter related to the tip penetration into the sample, called deformation and E* is the reduced elastic modulus. The adhesion force, E*_,_ and the deformation were obtained from the Force-Distance traces of the PF-QNM nanoindentations. E* is related to the Young’s modulus of the sample by its Poisson’s ratio which we fixed as 0.3 for both PTT and PTT-WS_2_ since we assumed the nanocomposite would have the same Poisson’s ratio as PTT. For more in-depth information see Appendix A. 

To extract information, we must know the spring constant of the cantilever, the radius of the tip and the deflection sensitivity of the cantilever. We calculate the spring constant using Sader’s method [61], and the radius of the tip and the deflection sensitivity of the cantilever are obtained using Bruker^®^ software and measuring standardized samples.

We used the aforementioned AFM device, but changed the tip to a RTESPA-300 (Bruker^®^) with a curvature radius of 13 nm, a nominal resonant frequency of 300 kHz, and a typical spring constant of 40 N/m. We carried out these measurements for samples at relative humidity (RH) and temperature values of 28 ± 1% and 21 ± 0.5 °C, respectively, as monitored by a temperature and RH data logger (EL-USB-2-LCD, Lascar Electronics, Whiteparish, UK).

## 3. Results and Discussion

### 3.1. Morphology

We have successfully produced LIPSS (LSFL) in a narrow fluence range from 15.9 to 31.3 mJ/cm^2^ for PTT, and from 19.1 to 33.9 mJ/cm^2^ for PTT-WS_2_. In both systems, LIPSS form parallel to the laser polarization with a period close to the wavelength of the laser. No ablation is taking place. In Figure S2, the AFM profile of the limit between a non-irradiated and an irradiated area shows that the peaks and valleys of the ripples lie, respectively, above and below the initial surface.

**Figure 1:**
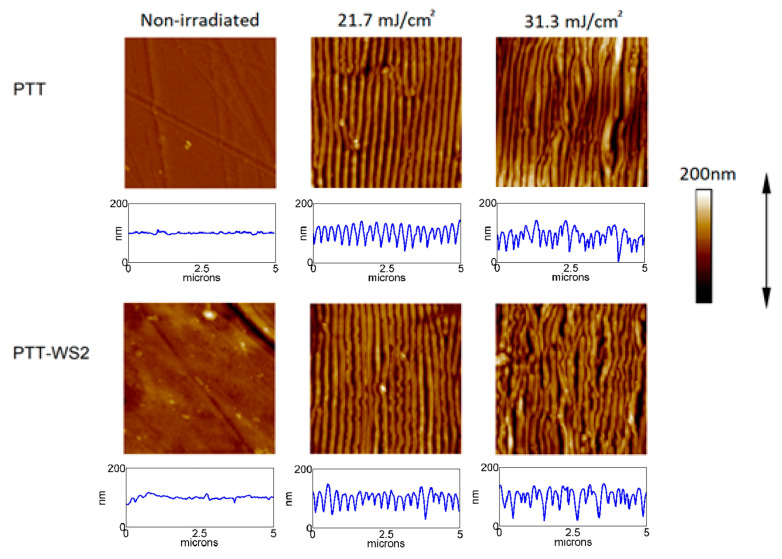
AFM images (5 × 5 μm^2^) and height profile over a five-micron horizontal line of PTT and PTT-WS_2_ samples after irradiation with 10,000 pulses with increasing fluences. The laser polarization follows the direction of the arrow.

Figure 1 displays a subset of AFM measurements which is representative of the evolution of the topography of the sample as the fluence of the pulses increases. The initial samples have low roughness—PTT R_a_ = 3 nm, PTT-WS_2_ R_a_ = 4 nm—and when we irradiated them with barely enough energy to trigger LIPSS formation, LIPSS of small length and depth start appearing, which grow longer and deeper as we increase the pulse fluence until we reach a certain threshold where LIPSS start losing periodicity and become disordered structures.

We can conclude that the presence of the nanoadditive leads to an increase in the energy density needed to trigger LIPSS formation. Since we know that T_g_ is the same for both samples as shown in Table 1, this could be caused by the higher thermal dissipation of PTT-WS_2_—from 9.6 × 10^−7^ in PTT to 10.6 × 10^−7^ in PTT-WS_2_—or its higher crystallization percentage from 30.1% in PTT to 32.1% in PTT-WS_2_. The thermal dissipation affects the LIPSS formation threshold, because the higher it is, the less efficient the laser heating, and, therefore, the more fluence and pulses are necessary to heat the sample. Regarding crystallization percentage, as explained in the introduction, crystallized polymers need to reach a temperature (T_m_), which is much higher than the one amorphous polymers need (T_g_) to be able to flow and form LIPSS, so an increase in crystallinity implies an increase in the temperature needed for LIPSS formation, and thus, more fluence and pulses are needed to reach it.

**Figure 2:**
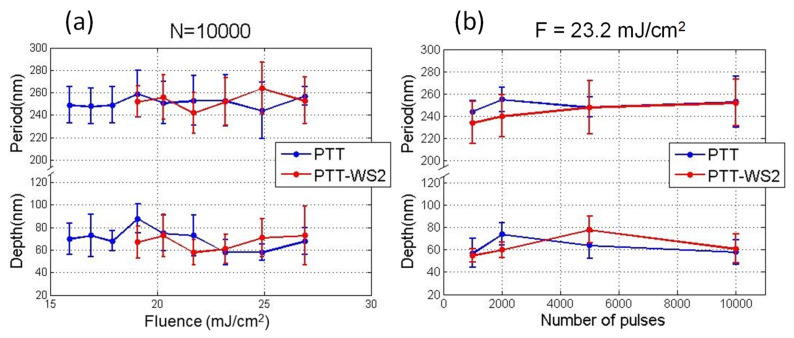
Dependence of LIPSS period and depth, on the number of pulses at a fluence of 23.2 mJ/cm^2^ per pulse (**a**) and on the fluence at 10,000 pulses per irradiation (**b**).

Figure 2 shows an example of the dependence of the period and depth of LIPSS on the number of pulses for a fixed fluence, and on fluence for a fixed number of pulses, respectively. When keeping constant the number of pulses, for the range of fluences at which LIPSS are observed, there is no clear dependence—neither of the period (with periods close to the laser wavelength) nor of the depth—and they remain almost constant. In contrast, when keeping the fluence constant, while the period remains almost constant with values close to the laser wavelength, the depth increases slightly with the number of pulses until it reaches a plateau. Similar dependence has already been reported for different polymeric materials [43] and can be explained attending to the mechanisms of LIPSS formation, which require, first, the polymer to be able to flow. If the polymer is amorphous, this can happen when the irradiation is enough for its temperature to increase above its T_g_, and segmental chain dynamics are allowed. If we keep increasing the fluence of the laser pulses, the maximum depth of the sample that is heated above T_g_ increases, and so, the depth of the structures grows. LIPSS dependence with the number of pulses is similar to their behavior with the fluence. This is a consequence of the well-known importance of the feedback and incubation mechanisms present in the formation of LIPSS [24].

### 3.2. Wettability and Surface Energy

Table 2 shows the average results of the measurements of CA with deionized water, glycerol and paraffin oil for raw and nanostructured PTT and PTT-WS_2_. Both PTT and PTT-WS_2_ are hydrophilic regardless of their surfaces being nanostructured or not. There are no significant differences between both materials, which could be expected given the percentage of additive is very small. For irradiated samples, the water CA decreases, indicating that the materials become more hydrophilic after irradiation. This could be explained assuming a homogeneous wetting model for water, Wenzel’s model [62]. This model assumes that resulting from viscosity and chemical interactions, the contact between water and the surface of the sample is not altered by the presence of air. It explains the variation of the contact angle as a function of a parameter called r. The r parameter is given by the relation between the total surface of solid in the solid-liquid interface, and the projection of the total surface of solid in the interface:

(3) $$r=\frac{\;total\;surface}{projected\;surface}$$

(4) $$cos\theta^{*}=r\;cos\theta$$

Therefore, since LIPSS formation increases the r parameter, if the original sample is hydrophilic it will become more hydrophilic, and if hydrophobic it will become more hydrophobic. However, for glycerol and paraffin oil, there are no significant changes in CA values after irradiation. 

Surface energies are presented in Table 3 and Table 4. The values obtained for the surface energy components differ depending on the method used for their calculation, mainly $\gamma^{p}$. In OWRK’s model, for both materials, $\gamma^{p}$ increases when the sample is irradiated, $\gamma^{d}$ slightly decreases, which adds up to a small increment of $\gamma^{TOT}$.

Meanwhile, in Van Oss, Chaudhury and Good’s model, for all the samples, $\gamma^{+}$ is much smaller than $\gamma^{-}$. This difference is even more prominent for irradiated samples, given the increase in $\gamma^{-}$ and the decrease in $\gamma^{+}$ after irradiation. Since $\gamma^{d}$ remains constant, $\gamma^{TOT}\;$ changes directly in relation to the changes in the polar components. There are no significant changes in the total surface energy of both samples.

The discrepancies can be attributed to the unreliability of OWRK’s model to calculate $\gamma^{p}$ when hydrogen bridges come into play (see Fowkes [63] and Panzer [64]) as it is the case when water is our probe liquid in the contact angle measurements.

In summary, the total surface energy does not change significantly from non-irradiated to irradiated samples. However, there is an important increase in the negative polar component of the irradiated samples. This cannot be explained by Wenzel’s model alone. It suggests the formation of polar hydrophilic species caused by a reaction with the oxygen in the air, catalyzed by the ionization and high temperature of the surface of the sample while the irradiation took place. Micro-Raman spectroscopy analysis of the irradiated films (see Figure S3) reveals a slight broadening of the band corresponding to the stretching vibration of the C=O bond (band at around 1722 cm^−1^), which is an indication of new carboxylic acid group formation. Similar results have been reported previously for the case of PET [6,65].

### 3.3. Adhesion and Elastic Modulus

**Figure 3:**
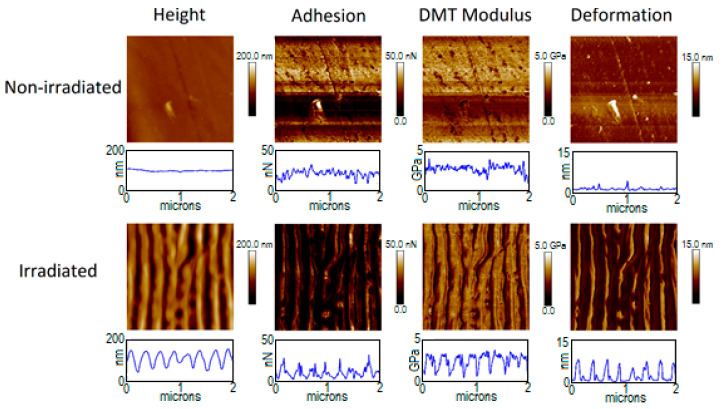
PF-QNM measurements of PTT surfaces before and after irradiation with 5000 pulses at 20.3 mJ/cm^2^. (2 × 2 μm^2^ images and property profiles over a 2 μm horizontal line).

**Figure 4:**
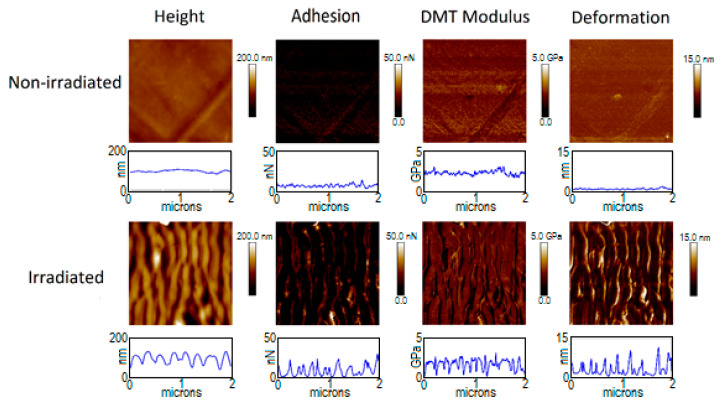
PF-QNM measurements of PTT-WS_2_ surfaces before and after irradiated with 5000 pulses at 20.3 mJ/cm^2^. (2 × 2 μm^2^ images and property profiles over a 2 μm horizontal line).

Figure 3 and Figure 4 show the mechanical properties of PTT and PTT-WS_2_ before and after irradiation. The non-irradiated samples present a very small roughness, as seen with the AFM measurements, and their adhesion, Young’s Modulus (where Equation (A2) of the appendix has already been applied to the values in the images) and deformation (shown in Table 5) are homogenous in the whole image. The irradiated samples, however, are topographically dependent, exhibiting nonhomogeneous values across the nanostructured surface. This effect is not due to the actual properties of the sample, but it is an effect derived from the way we are measuring them. When we adjusted the radius of the tip, as it was explained before, it is not considered that the sample surface might be curved, so the measurements give only real values for plane surfaces. Therefore, reliable values of the mechanical properties must be taken from the crests and valleys of the LIPSS, as in these zones the contact between the tip and the material could be considered equivalent to the normal contact of the tip with a planar surface, in contrast with the contact with the walls of the nanostructures. Following this criterion, we obtain the results in Table 5.

The measurements for the non-irradiated systems agree with previous characterizations [9]. For the irradiated samples, we found that there are no significant changes in the values of the elastic modulus, but the adhesion force decreases in a factor of ~4. The decrease of adhesion force after surface nanostructuring has already been reported [5,66]. However, that decrease was attributed to a smaller contact surface between the sample and the measuring device, as a result of the new surface morphology. Since in our experiment, the spatial resolution of our measuring technique—PF-QNM—is smaller than the size of the LIPSS, this decrease in adhesion cannot be explained on the same basis. Together with the magnitude of the contact surface between tip and sample, according to Dupré’s equation [67], the adhesion force between two surfaces is given by their respective surface and interaction energies. Since the tip is always the same standardized one, a change in adhesion implies modifications either on the total surface energy of the sample or on its interaction with the tip. From the contact angle measurements, we know that although there is no appreciable change in the total surface energy, there is an important decrease in the polar positive component as well as an important increase in the polar negative component. Although a combination of several intermolecular interactions and phenomena influencing adhesion should be considered—factors such as van der Waals or dipole-induced forces, electrostatic forces, H-bonding and capillary forces—electrostatic and H-bonding interactions represent a major part of the molecular forces acting between both contact surfaces. Considering that, the negative polar component of the surface increases after irradiation and that silicon surface might be negatively charged easily, electrostatic repulsion may play the most relevant role in this case.

Additionally, if we consider that the surface becomes more hydrophilic, low values of adhesion have been obtained before for –OH coated surfaces [68] and this was explained considering that high polarity of this tail group confers a high reactivity especially with contamination in air, and similar for –COOH groups. 

Furthermore, similar results to the ones reported by us have been previously observed by repetitive UV laser irradiation of PMMA at conditions below ablation and are related to the UV photomodification of the polymer [69] 

## 4. Conclusions

We have induced LIPSS with UV femtosecond laser pulses in both PTT and PTT-WS_2_ surfaces. In all cases, the nanostructures are parallel to the polarization of the incident laser. The period of the structures is around 260 nm, close to the laser wavelength. 

LIPSS emerge for fluences below the ablation threshold of the materials, from 15.9 to 31.3 mJ/cm^2^ for PTT, and from 19.1 to 33.9 mJ/cm^2^ for PTT-WS_2_, conditioned by the number of pulses (500–10,000). From this data, we can conclude that the presence of the nanoadditive leads to an increase of the energy density needed to trigger LIPSS formation. We explain this as the effect of the higher crystallization percentage and thermal dissipation of PTT-WS_2_.

The behavior of LIPSS period and depth with the fluence and number of pulses is similar to that reported for other polymers and was explained turning to the formation mechanism of LIPSS in polymers and the importance of feedback and incubation in the generation of LIPSS.

We have studied the wettability and surface energy of the samples, finding that the former increased with the formation of LIPSS and the total surface energy remained constant. However, its negative polar component increased heavily. This suggests the formation of polar hydrophilic species, caused by a reaction with the oxygen in the air, catalyzed by the ionization and high temperature of the surface of the sample while the irradiation took place. 

We characterized the topography and mechanical properties of the sample, finding that the formation of LIPSS did not change the Young’s modulus remarkably, but it induces a decrease of the adhesion force in both materials by a factor of four. We attribute this effect to the change of surface chemistry, as also indicated by the contact angle measurements.

In conclusion, LIPSS emerged at slightly higher energies for the nanocomposite than for raw PTT but produced almost equal effects in both PTT and PTT-WS_2_. Therefore, LIPSS surface nanostructuring can be used in this nanocomposite without any demerit. Hence, we can use LIPSS to easily change the surface properties of the nanocomposite, specifically the mechanical ones. Moreover, given the high control over the nanostructured area, we could create small zones with different nanostructures and thus, different surface properties.

## Figures and Tables

**Table 1:** Thermal properties of PTT and nanocomposite (PTT-WS_2_) [9]: T_m_ (melting temperature), T_g_ (glass transition temperature), ∆C_p_ (heat capacity), T_c_ (crystallization temperature) and X_c_ (mass percentage of crystallinity); and K (thermal conductivity) and α = $K/{\Delta C}_{p}\rho$ (thermal diffusivity) obtained from the PTT data [43] and the properties of WS_2_ nanotubes applying the rule of mixtures, an approach that has proven to be effective for other nanocomposites [54].

Sample	T_m_ (°C)	T_g_ (°C)	∆C_p_ (J/g °C)	T_c_ (°C)	X_c_ (%)	K (W(m K)^−1^)	α (m^2^ s^−1^)
PTT	229	53	0.17	171	30.1	0.22	9.6 × 10^−7^
PTT-WS_2_	228	53	0.16	177	32.1	0.23	10.6 × 10^−7^

**Table 2:** Average contact angles measured by means of the sessile drop technique with deionized water, glycerol and paraffin oil for non-irradiated samples and samples irradiated with 5000 pulses and a fluence of 20.3 mJ/cm^2^.

Sample	Deionized Water	Glycerol	Paraffin Oil
PTT	64°±1°	58°±5°	12°±1°
PTT LIPSS	44°±4°	57°±3°	12°±4°
PTT-WS_2_	67°±3°	61°±5°	17°±5°
PTT-WS_2_ LIPSS	50°±4°	58°±3°	16°±2°

**Table 3:** Values of the polar $(\gamma^{p}$), dispersive ($\gamma^{d}$) and total ($\gamma^{TOT}$) contributions to the surface energy for non-irradiated samples and samples irradiated with 5000 pulses and a fluence of 20.3 mJ/cm^2^. Calculated according to the WORK’s model (mJ/m^2^).

Sample	$\gamma^{p}$	$\gamma^{d}$	$\gamma^{TOT}$
PTT	14±1	27±1	41±1
PTT LIPSS	27±3	23±3	50±4
PTT-WS2	13±1	26±1	39±1
PTT-WS2 LIPSS	23±2	24±2	47±3

**Table 4:** Values of the polar positive ($\gamma^{+}$), polar negative ($\gamma^{-}$), dispersive ($\gamma^{d}$) and total ($\gamma^{TOT}$) contributions to the surface energy for non-irradiated samples and samples irradiated with 5000 pulses and a fluence of 20.3 mJ/cm^2^ according to the van Oss, Chaudhury and Good’s model (mJ/m^2^).

Sample	$\gamma^{+}$	$\gamma^{-}$	$\gamma^{p}$	$\gamma^{d}$	$\gamma^{TOT}$
PTT	1.59±0.07	17±1	10.5±0.5	28.2±0.2	38.7±0.5
PTT LIPSS	0.40±0.04	47±2	8.7±0.5	28.2±0.4	36.7±0.7
PTT-WS_2_	1.32±0.08	17±3	9±1	27.6±0.7	37±1
PTT-WS_2_ LIPSS	0.57±0.05	39±5	9.4±0.7	27.9±0.3	37.3±0.8

**Table 5:** Measurements of the deformation, adhesion and Young’s Modulus (E) obtained with PF-QNM for non-irradiated samples and samples irradiated with 5000 pulses and a fluence of 20.3 mJ/cm^2^.

Sample	E (GPa)	Adhesion (nN)	Deformation (nm)
Non-irradiated PTT	3.0 ± 0.5	24.0 ± 8.0	2.1 ± 0.8
Irradiated PTT	3.3 ± 0.2	6.8 ± 1.3	0.5 ± 0.2
Non-irradiated PTT-WS_2_	2.4 ± 0.2	9.0± 2.0	1.3 ± 0.3
Irradiated PTT-WS_2_	2.3 ± 0.2	2.2 ± 0.7	2.1 ± 0.4

**Figure A1:**
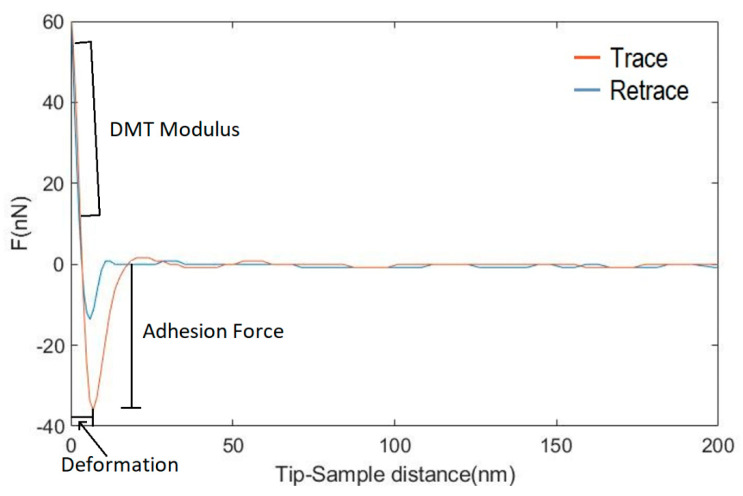
Force-distance trace and retrace of a PF-QNM nanoindentation in a PTT sample before being irradiated. The characteristics of the curve from which the different nanomechanical properties are obtained have been pointed out.

## Supplementary Materials

The following are available online at https://www.mdpi.com/2073-4360/12/5/1090/s1, Figure S1: UV-Vis absorption spectra of PTT, Figure S2: AFM image and corresponding profile at the boundary of an irradiated area of PTT, Figure S3: Micro-Raman spectra of PTT before and after irradiation.

## Appendix A. Calculation of Nanomechanical Properties from the Force-Distance Curves of PF-QNM

The adhesion force, *E^*^*, and the deformation were obtained from the maximum negative force, the slope of the linear part of the force-distance curves, and the distance from the point where the deformation stops being linear (deformation force level) to the point of peak interaction force respectively (Figure A1). We chose the limits of the linear part as 90% and 20% of the peak force for the maximum and minimum values. In this fitting range, both trace/retrace curves superimposed, which means we are working in the elastic regime and the use of the DMT model is justified. *E** is related to the Young’s modulus of the sample by:

(A1) $$\frac{1}{E^{*}}=\frac{1-\nu_{sample}^{2}}{E_{sample}}+\frac{1-\nu_{probe}^{2}}{E_{probe}}$$

where *v*_sample_ and *v*_probe_ are the Poisson’s ratios of the sample and probe respectively. As the sample’s Young’s modulus (*E*_sample_) may be considered much smaller than the probe’s one (*E*_probe_), the second term in the right side of Equaton (A1) can be neglected and we can calculate the elastic modulus of the sample as:

(A2) $$E_{sample}=\left(1-\nu_{sample}^{2}\right)E^{*}$$

where *v*_sample_ is fixed to a constant value of 0.3.

**Table A1:** Surface energy components of sample liquids (mJ/m^2^) [57].

Liquid	$\gamma_{l}^{d}$	$\gamma_{l}^{-}$	$\gamma_{l}^{+}$	$\gamma_{l}^{p}$	$\gamma_{l}^{TOT}$
Deionized Water	21.8	25.5	25.5	51.0	72.8
Glycerol	34.0	57.4	3.92	30.0	64.0
Paraffin oil	28.9	0	0	0	28.9
