# Supplementary material for: Laser-Induced Periodic Surface Structuring of Poly(trimethylene terephthalate) Films Containing Tungsten Disulfide Nanotubes
Source: Polymers (Basel). 2020 May 10;12(5):1090. doi: 10.3390/polym12051090 (PMC7284604; doi:10.3390/polym12051090)
Supplement: Supplementary file 1 [file polymers-12-01090-s001.pdf]

Supplementary Information

Laser-Induced Periodic Surface Structuring of Poly(trimethylene terephthalate) Films Containing Tungsten Disulfide Nanotubes

Javier Prada-Rodrigo [1,2,*], René I. Rodríguez-Beltrán [1,3], Sandra Paszkiewicz [4], Anna Szymczyk [5], Tiberio A. Ezquerra [6], Pablo Moreno [1] and Esther Rebollar [2,*]

[1] Grupo de Aplicaciones del Láser y la Fotónica (ALF-USAL), Universidad de Salamanca, Pl. de la Merced s/n, 37008 Salamanca, Spain; rrodrigu@cicese.mx (R.I.R.-B.); pmoreno@usal.es (P.M.)

[2] Instituto de Química Física Rocasolano, Consejo Superior de Investigaciones Científicas (IQFR-CSIC), Serrano 119, 28006 Madrid, Spain

[3] CONACYT- Unidad Foránea Monterrey, Centro de Investigación Científica y de Educación Superior de Ensenada, Alianza Centro 504, PIIT, Apodaca, Nuevo León CP 66629, México

[4] Department of Materials Technology, Faculty of Mechanical Engineering and Mechatronics, West Pomeranian University of Technology, Piastow Av. 19, PL-70310 Szczecin, Poland; spaszkiewicz@zut.edu.pl

[5] Department of Technical Physics, Faculty of Mechanical Engineering and Mechatronics, West Pomeranian University of Technology, Piastow Av. 19, PL-70310 Szczecin, Poland; anna.szymczyk@zut.edu.pl

[6] Instituto de Estructura de la Materia, Consejo Superior de Investigaciones Científicas (IEM-CSIC), Serrano 121, 28006 Madrid, Spain; t.ezquerra@csic.es

* Correspondence: javierprada@usal.es (J.P.-R.); e.rebollar@csic.es (E.R.)

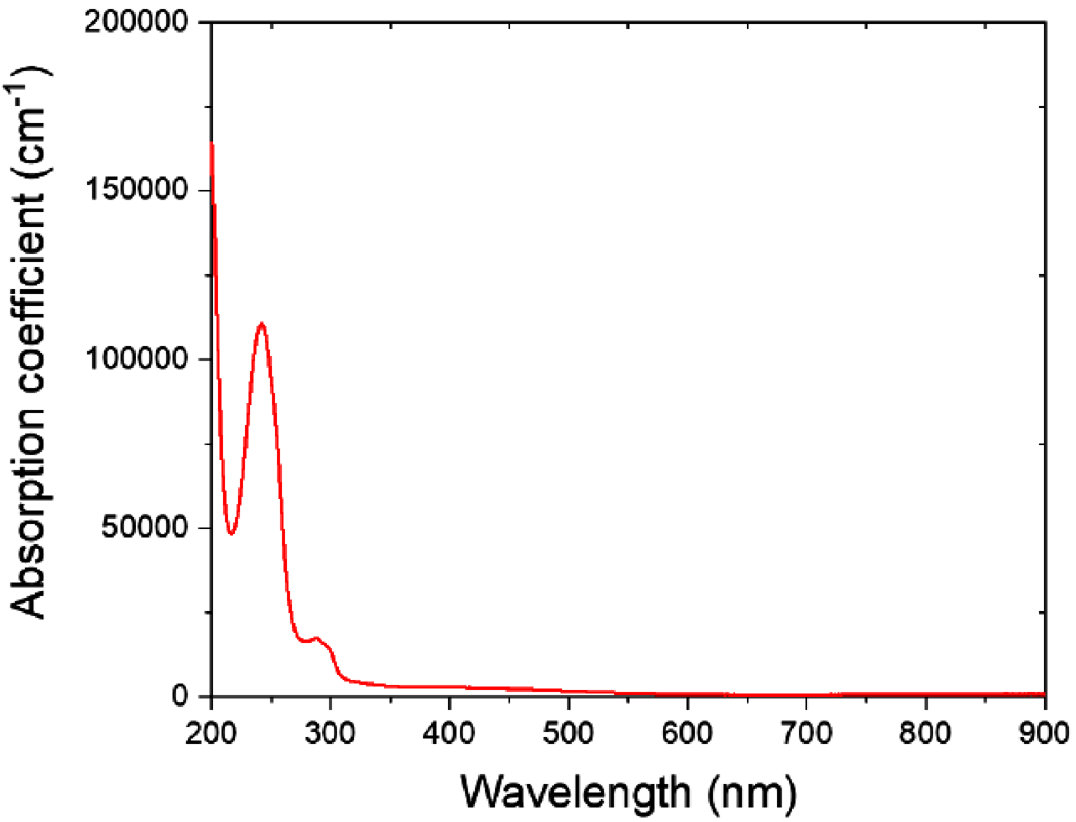

Figure S1. UV-Vis absorption spectra of PTT.

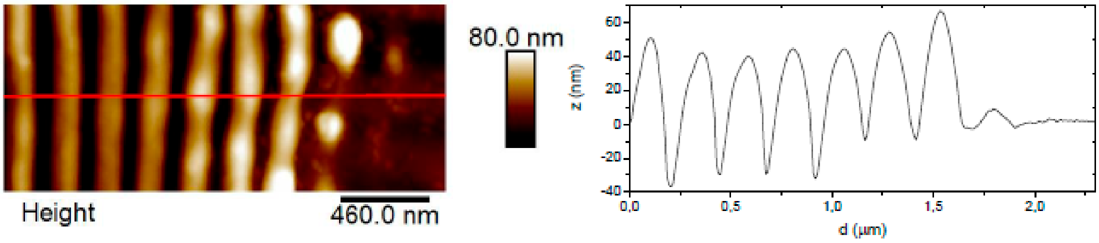

Figure S2. AFM image and corresponding profile at the boundary of an irradiated area of PTT.

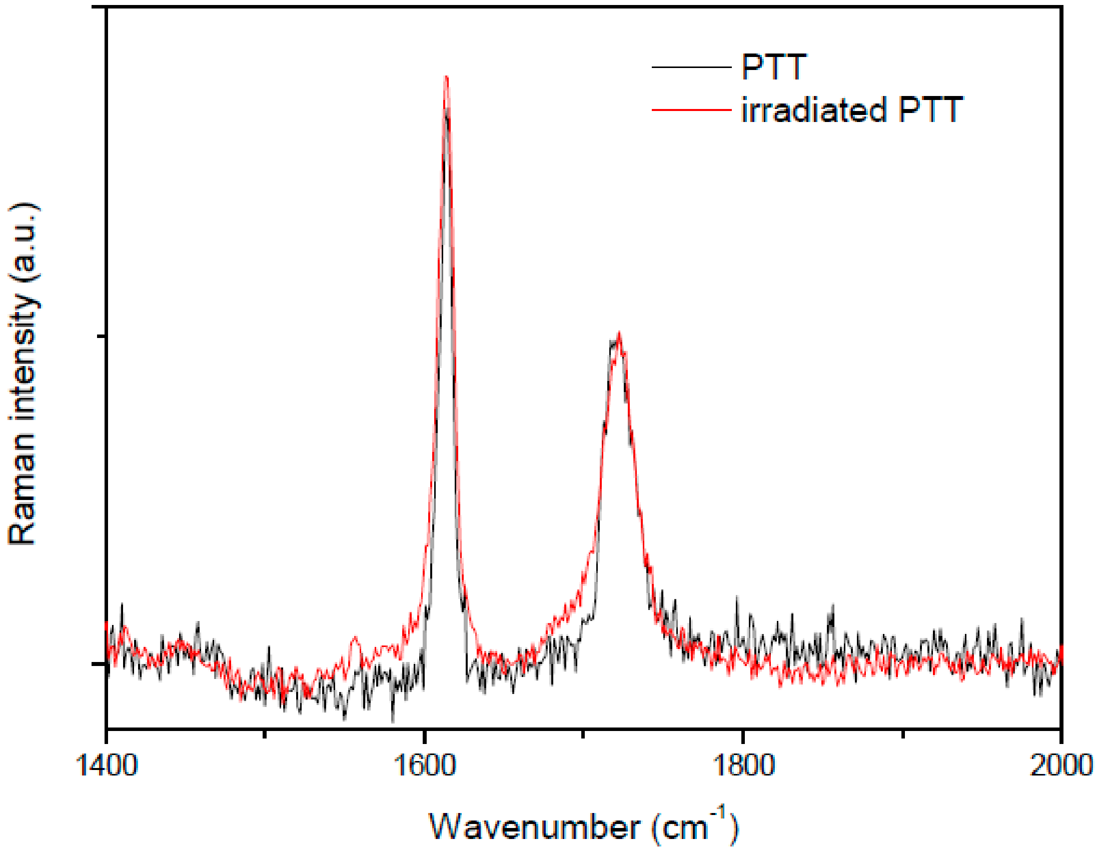

Figure S3. Micro-Raman spectra of PTT before and after irradiation.
